# Supplementary material for: Digital cloning of online social networks for language-sensitive agent-based modeling of misinformation spread
Source: PLoS One. 2024 Jun 21;19(6):e0304889. doi: 10.1371/journal.pone.0304889 (PMC11192300; doi:10.1371/journal.pone.0304889)
Supplement: S1 File — (ZIP) [file pone.0304889.s001.zip › Supporting Information.docx]

Supporting information

# **S1 Appendix**

# Community labels were extracted by leveraging the BERTopic library to apply a class-based term-frequency in-verse-document-frequency (c-TF-IDF) technique to a random sample of ~10,000 tweets from each community. After performing the topic modeling for each community, the c-TF-IDF labels from the top two most popular topics were merged together by our research team to form the final, human-comprehendible labels for each community. The identified communities along with their labels and populations are displayed in Table S1A.

**S1A Table. Network communities and populations**

| **Community** | **Label** | **Population** | **No. retweeted Period I-III posts** |
| --- | --- | --- | --- |
| 0 | Pharmaceutical company conspiracies | 3780 | 1,098,320 |
| 1 | UK politics | 2695 | 1,371,732 |
| 2 | Trump/Biden | 934 | 313,308 |
| 3 | Social distancing | 904 | 456,131 |
| 4 | Assange | 756 | 322,265 |
| 5 | Canadian politics | 736 | 309,400 |
| 6 | Other | 173 | 52,017 |

In Table S1B, presented below, we also present a set of network statistics for *N_A_,* the subnetwork used a basis for all network clone experiments discussed in the main text. Within the table, we present the 5%, 50%, and 95% quantile values for each metric, with the metrics reported to a maximum of three significant digits. Additionally, the edge density of our subnetwork is 0.005.

**S1B Table. Subnetwork statistics**

| **Metric** | **5% quantile value** | **50% quantile value** | **95% quantile value** |
| --- | --- | --- | --- |
| In-degree | 0 | 9 | 144 |
| Out-degree | 1 | 24 | 192 |
| Clustering coefficient | 0.03 | 0.21 | 0.44 |
| Eigenvector centrality | 0.000 | 0.002 | 0.017 |
| Closeness | 0 | 0.37 | 0.45 |

# **S2 Appendix**

# Our mutation model was built as follows. First, we identify the set of users within *N_A_* who authored more than 25 quote tweets in Period I-II and more than 20 recorded quote tweets in Period III. The quote tweets from Period I-II are used as prompt context for mutation prediction, while the tweets from Period III are used for mutation model evaluation. These filters select for users with data footprints large enough to conduct both processes effectively. Second, we engineer a gpt-3.5-turbo LLM prompt to ingest a users’ QT history, as well as a parent tweet (PT) of interest, and produce an AC prediction (LLM-AC). The QT history consists of a list of (PT, AC) pairs sampled from Period I-II for each user. While the LLM is leveraged to predict the results of a user QT, it is not leveraged to predict whether a user will respond to a PT with a QT or a RT. To this end, within our SEI ABM framework, a random draw based on a users’ Period I-II QT:RT frequency count ratio determines whether a user exposes his followers to a mutated (QT) or un-mutated (RT) strain of their infection.

# For each Period III evaluation event, PT and AC are recorded, the latter of which is compared to the LLM-AC. In Fig. S2A, we compare the cosine similarity between BERT embeddings of AC and LLM-AC. We focus on BERT embeddings since they serve as the foundation for the infection model discussed in Section 2 of the main text and consequently are relevant for infection probability calculations.

# For each evaluation item, we also construct the following three strings:

# i) LLM-AC + PT (LLM prediction)

# ii) AC + PT (ground truth)

# iii) PT (naïve baseline)

# Here, (iii) represents a no-mutation naïve baseline model. Since users exhibit varying levels of predictability, within our ABM, we only enable mutations for users where the average Period III cosine similarity between (i)-(ii) embeddings is greater than the average cosine similarity between (i)-(ii) embeddings.

**S2 Fig. Mutation model evaluation.**

**(A)** Histogram of cosine similarities calculated between mutation model AC prediction embeddings and AC ground truth embeddings within our evaluation set. **(B)** Cosine similarity histograms between AC + PT prediction embeddings and AC + PT ground truth embeddings for users who satisfied the mutation model criteria specified in Section X. AC + PT is calculated both using our mutation model and a static baseline in which AC is always set to an empty string, for comparison purposes. The mutation model offers slightly higher similarity to the ground truth data than the baseline, suggesting more accurate infection probabilities can be calculated when accounting for mutations.

# For these users, we compute the cosine similarity between BERT embeddings of the (i)-(ii) and (i)-(iii) pairs in Fig S2B. The modest improvement in cosine similarities produced by (i) relative to (iii) suggests modeling mutation through a LLM yields more accurate embeddings than ignoring such events for the subset of users we studied. Combined, Fig S2A and Fig S2B demonstrate the ability of LLM’s to reproduce AC’s for a select set of users and the extent to which this capability yields mores realistic QT embeddings.

# We explored performance across varying LLM temperatures and user quote history context lengths and found that lower temperatures and higher context lengths resulted in more accurate performance. Context length was limited by the size of our dataset in Period I-II and by LLM prompt token limits. The number of users who matched the data filters listed above were less than <1% of total network users; however, expanding the time period of Period I-II and shifting to longer context window models will increase the number of eligible users. While due to these limitations, our mutation model had relatively minor impacts on ABM infection rates, we believe the proof-of-concept work presented here may open up additional investigation of information mutation. For example, the information propagation mechanism explored in this work is post reshares; however, users often transmit information through other means. For examples users may ingest information from their social network and, instead of a reshare, produce an original post inspired by these thoughts. Further work can explore how these events may be simulated through similar protocols as above.

# **S3 Appendix**

In the Table S3 below, we present the post text for all posts used to construct Fig 8C in the main text. As the only post extracted from real user activity, the text for ‘Vaccines’ has been modified to protect individual privacy. The remaining posts were generated synthetically.

| **Post Topic** | **Post Text** |
| --- | --- |
| Vaccines | Trying to pretend that knocking out an experimental #vaccine in 6 months is normal' and 'completely safe'. Absolute liars, Media are criminal |
| Cooking | Check out my new recipe for chicken soup! |
| COVID | Just heard from a friend in the medical field that the vaccine has microchips to track us. Don't be sheep! #COVIDLies #WakeUp |
| Elections | Overheard at a coffee shop that there were thousands of fake ballots found in the last election. The system is rigged! #ElectionFraud |
| Global warming | So we had a cold day in July, and they still want us to believe global warming is real? LOL. #ClimateHoax |
| Secret societies | Did you know top politicians meet in secret societies to control the world? Do your research! #DeepStateTruth |
| 5G | My cousin's friend got sick right after a 5G tower was installed near his house. Coincidence? I think not. #5GKills |
| GMO | Why eat organic when it's all a scam? GMOs are just as natural and safe. #OrganicMyth |
| Flat Earth | Read an article that says the Earth might actually be flat. Makes you question everything we've been told! #FlatEarthRevealed |
| Equal rights | Heard that these so-called 'equal rights' movements are just schemes to get more money and power. Don't be fooled! #RightsScam |

**S3 Table. Text for posts presented in Fig 8C**
