# Supplementary figures and images for: Digital cloning of online social networks for language-sensitive agent-based modeling of misinformation spread
Source: PLoS One. 2024 Jun 21;19(6):e0304889. doi: 10.1371/journal.pone.0304889 (PMC11192300; doi:10.1371/journal.pone.0304889)

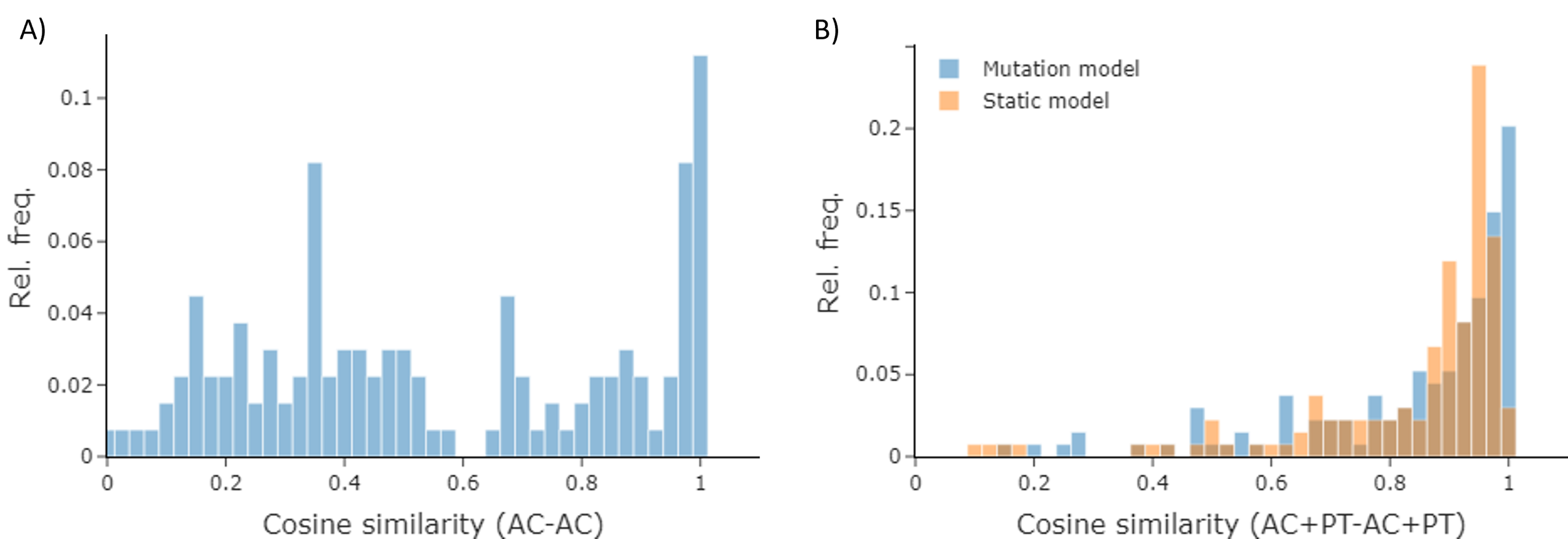

Supplement: S1 File — (ZIP) [file pone.0304889.s001.zip › S2_Fig.tif]
