# Supplementary material for: Digital cloning of online social networks for language-sensitive agent-based modeling of misinformation spread
Source: PLoS One. 2024 Jun 21;19(6):e0304889. doi: 10.1371/journal.pone.0304889 (PMC11192300; doi:10.1371/journal.pone.0304889)
Supplement: Attachment — Submitted filename: Rebuttal Letter.docx [file pone.0304889.s002.docx]

PLOS ONE

San Francisco, CA

April 26, 2024

Title:	**Digital cloning of online social networks for language-sensitive agent-based	modeling of misinformation spread**

Authors:	**Prateek Puri, Gabriel Hassler, Sai Katragadda, and Anton Shenk**

Article Type:	**Research Article**

Dear Editor:

Thank you for the thoughtful and careful consideration of our manuscript. Both you and the reviewers made discerning observations about our research, and we are grateful for the feedback. We have performed additional analysis, made modifications to the figures, and adjusted the manuscript text to reflect this feedback, and we believe our research product has become much stronger and more complete as a result.

Please see our responses to each point raised in our initial review. Under the relevant reviewer/editor comment, we have included passages from the manuscript text that have been modified. A subset of these excepts contain a mixture of modified and un-modified text. In most of these cases, we bold the modified text, for clarity. Thank you, and we are look forward to your response.

Best,

Prateek Puri

**Editor Comments**

1. “Please ensure that your manuscript meets PLOS ONE's style requirements, including those for file naming. The PLOS ONE style templates can be found at

https://journals.plos.org/plosone/s/file?id=wjVg/PLOSOne_formatting_sample_main_body.pdf and

https://journals.plos.org/plosone/s/file?id=ba62/PLOSOne_formatting_sample_title_authors_affiliations.pdf”

Thank you for the reminder of PLOS ONE’s style guide. We have modified the section headings to have the proper capitalization, adjusted the header indentations, removed the numerical prefixes to the section headings, added supporting information legends to the end of the manuscript, and modified how the supporting information is referenced within the main text.

We also adjusted the figure/table titles in the supporting information to match the style guide, and we also changed the file naming of both our supporting information and manuscript figures to match the specified convention. Lastly, we modified the title and authorship page to match the format in the link provided.

2. “Please provide additional details regarding participant consent. In the ethics statement in the Methods and online submission information, please ensure that you have specified what type you obtained (for instance, written or verbal, and if verbal, how it was documented and witnessed). If your study included minors, state whether you obtained consent from parents or guardians. If the need for consent was waived by the ethics committee, please include this information.

Once you have amended this/these statement(s) in the Methods section of the manuscript, please add the same text to the “Ethics Statement” field of the submission form (via “Edit Submission”).”

Our Human Subjects Protection Committee determined additional consent was not required from X users given the data sharing agreements X users opt into when creating public profiles. We have updated the text in the manuscript as well as in the “Ethics statement” of the online submission form to read as:

“We only extracted public posts which users, by agreeing to X’s data privacy terms and conditions, agreed to make broadly publicly accessible. The RAND Corporation Human Subjects Protection Committee (HSPC) reviewed and approved the data collection and handling protocols within this project. Given no private posts were obtained during this work, and given X’s data privacy policy, the HSPC board determined additional consent was not required from the studied users.”

3) “Before we proceed with your manuscript, please address the following prompts:

a) If there are ethical or legal restrictions on sharing a de-identified data set, please explain them in detail (e.g., data contain potentially identifying or sensitive patient information, data are owned by a third-party organization, etc.) and who has imposed them (e.g., a Research Ethics Committee or Institutional Review Board, etc.). Please also provide contact information for a data access committee, ethics committee, or other institutional body to which data requests may be sent.”

We understand the need for transparency of the datasets leveraged within this work and thank the editor for raising attention to this point.

Our reasons for not including the raw social media data analyzed in this work are two-fold. Firstly, these datasets were obtained through the Brandwatch platform, a third-party social media data vendor. According the Brandwatch data sharing agreement, only authorized users may access data extracted from the platform. While we are authorized to access Brandwatch data, we are not permitted to share this data publicly (a full set of their legal terms and conditions are available [here](https://www.brandwatch.com/legal-documents/legal/)). According to Brandwatch’s privacy statements and agreements, these protections exist to safeguard their proprietary data product and to ensure sensitive user information is only accessed by authorized users in contexts aligned with Brandwatch’s data ethics.

Secondly, the raw data from Brandwatch includes information such as user account id and Tweet post text, fields which both can be used to identify the specific accounts studied in this report. As part of our Human Subjects Protection Committee (HSPC) approval process (the entity at our institution responsible for IRB review), we stipulated such information would not be released publicly during our project. One alternative is to release datasets without post id and user id present. However, post text was the single most direct input to our infection and mutation models, meaning releasing a dataset where this field has been redacted would not allow other researchers to replicate our findings. The contact email for our HSPC board is [hspcadmin@rand.org](mailto:hspcadmin@rand.org) and the relevant point of contact is Carolyn Tschopik, the board member most actively involved in our review.

However, despite these restrictions, we still provide all raw data outputs from our anonymized ABM simulations so other researchers may better understand and replicate our findings. These datasets can be found at the following public data repository: <https://dataverse.harvard.edu/dataset.xhtml?persistentId=doi:10.7910/DVN/O17AWX>

If other researchers are interested in accessing the raw Brandwatch social media data, one option is for them to sign up for a Brandwatch consumer research account: <https://www.brandwatch.com/products/consumer-research/>

Since the editor mentioned that the data availability statement within our manuscript would be updated on our behalf after reading our response, we have not made any major changes to the manuscript or the submission forms to communicate this information. However, we updated the statements in our manuscript’s “Ethics statement” section to read as follows for greater clarity:

“However, a user id can be linked to a user’s profile, where, in certain cases, users may opt to share personally identifiable information publicly. Additionally, Brandwatch’s policies only permit authorized users to access data extracted on their platform. For both reasons, we do not release any of the raw social media data we analyzed in this work. However, we do present the raw data from our anonymized ABM simulations within a public data repository.”

4. “We notice that your supplementary figure is uploaded with the file type 'Figure'. Please amend the file type to 'Supporting Information'. Please ensure that each Supporting Information file has a legend listed in the manuscript after the references list.”

Thank you for drawing attention to this oversight. We have updated the file type and added the required figure legends to the end of the manuscript.

**Reviewer 1 Comments**

1. “I would suggest emphasizing more these outcomes in terms of the model's validity and their implications from an interpretative point of view in misinformation countermeasure evaluation.”

We thank the reviewer for drawing attention to the need to contextualize our findings within the broader scope of our research objectives. We have added the following text to the “Infection model” section to conveys the significance of the persistence of the infection model performance over time:

“We observe a degree of overfitting between the training and test sets; however, we notice only very slight performance degradation across time, suggesting Period I-II user behavior encoded during the training process remains relevant to user information sharing tendencies for multiple months (Fig 5B). **The persistence of infection model performance bodes well for the maintainability of simulation frameworks leveraging the model. For example, if performance degraded sharply over time, the model would need to be retrained frequently to produce infection probabilities aligned with current user preferences, imposing significant model maintenance costs. The lack of such degradation implies an infection model, once trained, may produce reasonably accurate infection probabilities across a time horizon spanning several months.”**

We also added the following text to the “Results” section to better frame the findings present within Figure 6:

“As shown in Fig 6A, the number of recorded infections within NA for type (i) posts demonstrates a reasonable correlation with that predicted by the ABM with a Pearson correlation in log-space equal to 0.81 (p < 0.01). A positive, albeit not statistically significant, correlation of 0.06 (p = 0.075) is observed (Fig 6B) for type (ii) posts. These results suggest most of the variation in virality explained by the ABM is attributable to user-level features; however, the ABM still does demonstrate a degree of text-sensitivity even when user-level features are fixed. For reference, static infection models that do not consider user or text-based features would not display any variation in virality across (i) and (ii) posts**. In summary, Fig 6A and Fig 6B demonstrate that post virality varies strongly both across users as well as across posts authored by a single user. This variation is partially reproduced by our dynamic ABM but is largely neglected by more traditional static infection probability frameworks, suggesting the tools explored here may help produce higher fidelity simulations of social network activity. Lastly, while Fig 6 suggests user-level features account for a majority of explained ABM variation, the infection model architecture can be adjusted to place more or less of an emphasis on text-based features, allowing for balancing of ABM text sensitivity with simulation fidelity.**

2. “Finally, I would add a column with the number of posts for each community in Table S1”

We adjusted Table S1A to include a column “No. retweeted Period I-III posts” that indicates the total number of posts authored by users from each community that were subsequently retweeted by other users. This table is populated with the updated communities obtained after resampling our network based on suggestions from Reviewer 2. The table is included below, for reference:

**S1A Table. Network communities and populations**

| Community | Label | Population | No. retweeted Period I-III posts |
| --- | --- | --- | --- |
| 0 | Pharmaceutical company conspiracies | 3780 | 1,098,320 |
| 1 | UK politics | 2695 | 1,371,732 |
| 2 | Trump/Biden | 934 | 313,308 |
| 3 | Social distancing | 904 | 456,131 |
| 4 | Assange | 756 | 322,265 |
| 5 | Canadian politics | 736 | 309,400 |
| 6 | Other | 173 | 52,017 |

**Reviewer 2 Comments**

1. “the paper uses a "cloned" network obtained starting from a post chosen by the authors. Although they explain why they chose that post, I think that it is necessary to add the analysis and the results for at least one other network to check the robustness and validity of the model”

We strongly agree with the reviewer that this work would benefit from additional checks to verify the generalizability of our results.

Unfortunately, partially due to recent data sharing policy changes at X, costs to accessing X data have risen substantially, and our research institution no longer has access to large-scale X datasets via Brandwatch. Without this data access, it is extremely difficult to rerun our experiment on a separate set of Twitter communities. Further, given the funding limitations of our non-profit organization, we do not have resources available to identify alternate, lower-cost pathways to obtaining such datasets, to the extent that they exist.

However, we do believe it is important to share the insights we learned from our initial X dataset with the research community, especially as access to X data has become more difficult in recent months for many researchers. We hope the work explored here will not only shed light on information sharing dynamics within X, a still popular social media platform, but also call attention to methods that may help explore dynamics within other social media platforms plagued by misinformation spread.

In summary, while we do not have the capacity, from a funding or data access perspective, to address this particular concern, we strongly agree with the remaining comments from Reviewer 2 and have carried out additional analysis and updated our manuscript to address them. Lastly, we added the following text to our “Discussion” section to highlight the importance of performing additional robustness checks to verify the generalizability of our results:

“However, there are several limitations of this work. Firstly, we evaluated our simulation system by replicating dynamics within a fixed set of X communities known to discuss COVID-19 conspiracy theories. Future work should reapply our framework to a different set of online communities and misinformation topics, either within X or a separate social media platform, to assess the generalizability of our results.”

2. “Line 119-120, the authors say they selected the 10000 most active users, measured with the in-degree of the node (i.e. how many times the user shares a post of another). My concern here is the following: consider a network in which node v has a high in-degree, but its neighbour is composed of user with low in-degree (similar to a star pointing to v). Thus, they are deleted in the filtered network G. In this way, node v is part of G, but its "activity" is highly underestimated because all its neighbour neighbours have disappeared. This is a possible problematic case that can arise but, in general, I think that the activity should be measured differently. Maybe both the in- and out-degree of nodes should be considered to measure activity. Moreover, I suggest to add some simple statistics of the network (edge density, degree distribution, etc.)”

We agree with the reviewer that we can improve our networking sampling procedure. We particularly appreciate the reviewer’s highlighting of the importance of including both information connectors (high in-degree) and information sourcers (high out-degree) within our subnetwork. We revised our sampling approach to follow the following process.

Firstly, we set subnetwork *N_A_* to be equal to *U_T_* (the set of users consisting of the source post user as well as all users who retweeted the source post). We then consider all other users in our dataset, not already a member of *N_A_*, and calculate the number of incoming posts (number of posts each user has retweeted that were authored by users within *N_A_*) and outgoing posts (number of posts authored by each user that users within *N_A_* have retweeted) to the subnetwork. We rank each user by their number of combined incoming and outcoming posts and add the highest ranked user to *N_A_*. We repeat this process until we have ~10,000 total users within our network. The sampling approach leverages a combination of one-hop neighbor sampling to first identify *U_T_* and then leverages a modified form of snowball sampling to iteratively identify users that interact most frequently with this subnetwork. For the latter step, the focus on both incoming and outgoing posts was meant to address the reviewer’s comment about balancing in-degree and out-degree connections.

We performed the sampling routine above, extracted an updated subnetwork, and repeated the entirety of our experiment with this updated subnetwork. Accordingly, we updated Figures 1, 5, 6, 7, an 8 as well as portions of the main text that discuss the results associated with these figures. We observe findings that are consistent with the first version of our paper, which was conducted with a different subnetwork sample. *In lieu* of the ability to repeat our experiment with a different source post (as discussed in the reply to Comment #1 by Reviewer 2), we believe the consistency of our findings across sampled subnetworks speaks, at least partially, to the robustness of our methods.

We updated the following portion the “Network Selection” section to describe the updated sampling procedure:

“To make our network size manageable for running simulations given available resources, we further narrowed the network via the following process. **Firstly, we define *N_A_* as the subnetwork to be used within our ABM and initially set *N_A_* = *U_T_*. We consider all users in *U_T_*, but not present in *N_A_*, and rank each user in this set, *u_i_*, according to the number of incoming** $\left( \sum_{\boldsymbol{j\epsilon}\boldsymbol{N}_{\boldsymbol{A}}} \boldsymbol{|}\boldsymbol{R}_{\boldsymbol{ji}}\boldsymbol{|} \right)$**and outgoing** $\left( \sum_{\boldsymbol{j\epsilon}\boldsymbol{N}_{\boldsymbol{A}}} \boldsymbol{|}\boldsymbol{R}_{\boldsymbol{ij}}\boldsymbol{|} \right)$ **posts they’ve participated in with users within *N_A_*, weighing each equally. Following a modified snowball sampling procedure, we add the highest ranked user to *N_A_* and repeat the process iteratively until we have 10,000 users in *N_A_* (S1 Appendix). Considering both the in-degree and out-degree connections of each user to *N_A_* during the subnetwork selection process helps balance including users who source information with those who disseminate information. “**

To the reviewer’s second point about presenting network statistics, we have updated Figure 1C to include both in-degree and out-degree network histograms. In addition, we updated the Supporting Information to report the measured subnetwork edge density (0.005). Lastly we added Table S1B to the Supporting Information, where we present the [5%, 50%, 95%] quantile values for the following subnetwork statistics: in-degree, out-degree, closeness, eigenvector centrality, and clustering coefficient.

3. “Line 136-137, the authors say that in Fig1A the edge thickness is proportional to the number of follower-followee between the two communities. However, in Line 121-122 it is written they didn't extract the relation followee-follower.
Could you explain better?”

We recognize the confusion and thank the reviewer for calling attention to this. A generic X user has a set of accounts that follow them or they follow themselves. However, information spreading pathways are not restricted to these relationships, i.e. a user may see and spread content from accounts they do not follow. For this reason, and also due to that fact that follower/followee information is difficult to extract from X’s platform, we define user relationships according to Equation 1 in the main text. To clarify this, we have adjusted the following passage in the “Network selection” section:

“**Lastly, on the X platform, each profile is associated with a set of users who follow the account and a second set of users the user of the account themselves follow.** **However, these relationships do not fully capture information spreading pathways.** We infer edges between nodes through Equation 1 rather than extracting followee 🡪 follower relationships from the X API for the two following reasons:

(1) Users are capable of retweeting information from individuals they do not follow. These information pathways are captured via the method above but are not captured by solely examining a user’s followees

(2) When this research was conducted, the X API has rate limits that would make such processing infeasible for our network

**For the remainder of the text, we will define follower and followee relationships as edge relationships determined by Equation 1 rather than those stated on a user’s profile.”**

We also added the following text to the “Community detection” section to more clearly explain the information within Fig 1A:

“Visualizations of these communities, as well as interactions between them, are presented in Fig 1A. In the figure, each node represents a community, where the node size (edge thickness) is proportional to the community size (number of total edges between users from separate communities)”

Lastly, we updated the caption of Fig 1 to additionally clarify the information within the network graphic:

“Fig 1. Base network characterization.

(A) Network diagram of our base social media network. Node size is proportional to community population number, and **edge thickness is proportional to the total number of user edges between two community nodes**. The labels are extracted by applying topic modeling to recorded tweet history within each community.”

4. “Line 168-189 (SEI Model Pseudocode), I found it hard to read due to the difficult-to-read notation. Moreover, it seems that some symbols are not defined. In particular:

1. Line 173: what is f(j)? Is t_j the time at which a user becomes infected? In case, please specify it better;

2. Line 177: I think you could simply say S(f_j) = S;

3. Line 179: (at least for me) it's very hard to read and interpret X(p = IP). Since it is a Bernoulli random variable,

please use a simpler notation;

In general, please check you defined everything you mention in the pseudocode.”

We thank the reviewer for pointing out this key oversight. We have updated our pseudocode to be more easily interpretable and have defined all referenced variables. Please see the revised text below:

“

***SEI Model Pseudocode***

I. source author ***u_S_*** is exposed to tweet ***T_s_***
   - set the state of user ***u_S_*** to exposed: S(***u_S_****)* 🡪 ***E***
   - initialize the set of exposed users: ***S_E_*** 🡪 *{****u_S_****}*
   - set author ***u_S_*** infection time: ***t_s_*** 🡪 0
   - set the originator of tweet: ***Orig(T_s_)*** 🡪 ***u_S_***
II. while |***S_E_****| > 0*:
    - Find the user ***u_i_*** with the lowest infection time *t_i_*
    - user ***u_i_*** is infective
      - S(***u_i_ )*** 🡪 ***I***
    - remove user ***u_i_*** from the set of exposed users
      - ***S_E_*** 🡪 ***S_E_*** *\* ***u_i_***
    - for each susceptible follower ***u_j_*** of ***u_i_*** (i.e., all ***u_j_*** such that ***u_j_*** follows ***u_i_*** and S(***u_j_)*** *=* ***S***)***:***
      - compute infection probability as ***IP*** 🡪 *IM*(***u_j_***, ***Orig(T_i_)*** ***, T_i_***) (***Orig(T_i_)*** is the originator of tweet ***T_i_***)
      - sample a uniform random variable: ***x_IP_*** *~ Uniform(0, 1)*
      - if ***x_IP_*** ≤ ***IP***:
        - follower ***u_j_*** is exposed: S(***u_j_ )*** 🡪 ***E***
        - follower ***u_j_*** is added to the set of exposed users: ***S_E_*** 🡪 ***S_E_*** ∪ *{****u_j_****}*
        - follower is assigned an infection time
          - sample ***Δ*** *~ Exponential(1)*
          - ***t_j_*** 🡪 ***t_i_*** *+* ***Δ***
        - compute quote tweet probability: ***QP*** 🡪 QM(***u_j_***)
          - QM(***u_j_***) is the empirical frequency at which user ***u_j_*** quote tweets (as opposed to retweets) from their observed twitter history. This quantity is pre-computed for each user.
        - sample ***x_QP_*** *~ Uniform(0, 1)*
        - if ***x_QP_*** ≤ ***QP***:
          - ***T_j_*** generated by LLM
          - ***Orig(T_j_)*** 🡪 ***u_j_***
        - if ***x_QP_*** > ***QP***:
          - ***T_j_*** 🡪 ***T_i_***
          - ***Orig(T_j_)*** 🡪 ***Orig(T_i_)***
      - if ***x_IP_*** > ***IP***:
        - *continue*

where S(***u_j_***) represents the SEI state of user ***u_j_***; ***S*** is the susceptible state; ***E*** in the exposed state; ***I*** in the infective state; *IM*(***u_j_***, ***u_k_, T_i_***) is a function that returns the probability of infection with features derived from the follower ***u_j_***, tweet’s source author ***u_k_***, and the tweet itself ***T_i_*** (see Infection Model section below). One thousand iterations of the above process are executed for each explored ABM scenario to capture stochastic variation, as shown for a sample event in Fig 5A.”

5. “Line 193: The authors say that \Delta is an arbitrary random variable, but I didn't find what type of distribution they use for it in the simulations. Please add some more details”

We thank the reviewer for highlighting this point of confusion. ***Δ*** is sampled from an exponential distribution. To improve clarity, we have updated the manuscript to define the variable ***Δ ,*** as well as the distribution from which it is sampled, within the pseudocode text itself (as shown in the manuscript excerpt within the above response).

6. ”Line 246: maybe you can add a citation to your sentence”

The reviewer makes an excellent suggestion. The reader will benefit from exposure to other research on the importance of quote tweets in shaping online discourse. We added the two references below to the part of the manuscript the reviewer flagged:

42. Wojcieszak M, Casas A, Yu X, Nagler J, Tucker JA. Most users do not follow political elites on Twitter; those who do show overwhelming preferences for ideological congruity. Science advances. 2022 Sep 30;8(39):eabn9418.

43. Zade H, Williams S, Tran TT, Smith C, Venkatagiri S, Hsieh G, Starbird K. To Reply or to Quote: Comparing Conversational Framing Strategies on Twitter. ACM Journal on Computing and Sustainable Societies. 2024 Jan 13;2(1):1-27.

Reference 1 describes a study where quote tweets were recognized as a key mechanism to broadcast messages across a user’s social network. Reference 2 discusses how quote tweets are often leveraged to promote particularly negative sentiments and opposition viewpoints, highlighting the role of quote tweets in amplifying online polarization.

7. “Line 318-319: the authors mention correlation values that are not shown. Please add them in the Figure or in a separate table, together with their significance (e.g. p-values). Moreover, please specify what coefficient did you use and if correlations are computed on logged values instead of original”

We agree with the reviewer that readers will benefit from more details on the measured correlation coefficients. We adjusted the caption of Figure 6 to include more details on the regression model we ran, and we also have updated the section of the manuscript where the results are discussed, as copied below:

“**As shown in Fig 6A, the number of recorded infections within *N_A_* for type (i) posts demonstrates a reasonable correlation with that predicted by the** **ABM with a Pearson correlation in log-space equal to 0.81 (p < 0.01). A positive, albeit not statistically significant, correlation of 0.06 (p = 0.075) is observed (Fig 6B) for type (ii) posts****.** These results suggest most of the variation in virality explained by the ABM is attributable to user-level features; however, the ABM still does demonstrate a degree of text-sensitivity even when user-level features are fixed. For reference, static infection models that do not consider user or text-based features would not display any variation in virality across (i) and (ii) posts. In summary, Fig 6A and Fig 6B demonstrate that post virality varies strongly both across users as well as across posts authored by a single user. This variation is partially reproduced by our dynamic ABM but is largely neglected by more traditional static infection probability frameworks, suggesting the tools explored here may help produce higher fidelity simulations of social network activity. **Lastly, while Fig 6 suggests user-level features account for a majority of explained ABM variation, the infection model architecture can be adjusted to place more or less of an emphasis on text-based features, allowing for balancing of ABM text sensitivity with simulation fidelity.”**

8. “At the end of the discussion section, please add a paragraph about the limitations of the work. Although you mentioned some of them throughout the paper, I think it could be useful to provide a summary in the end.”

We wholeheartedly agree with the reviewer that the reader would benefit from a summary of the limitations of this work. To this end, we have added the following paragraph to the Discussion section of the manuscript:

“However, there are several limitations of this work. Firstly, we evaluated our simulation system by replicating dynamics within a fixed set of X communities known to discuss COVID-19 conspiracy theories. Future work should reapply our framework to a different set of online communities and misinformation topics, either within X or a separate social media platform, to assess the generalizability of our results. Similarly, our ABM was built upon a simple SIR model that neglected more complex user interactions, such as refutation and debunking, known to influence propagation dynamics [49-50]. Further, we assumed a user exposed all followers to a given retweet; however, X’s recommendation algorithm plays a vital role in determining the posts each user views, a mechanism that has been modeled elsewhere [51]. Lastly, as described in the above Network selection section, subnetwork generation required removing a set of relationships known to exist within the base network. This process resulted in base and cloned networks with non-identical graph structures, ultimately hampering the fidelity with which the cloned network could replicate base network dynamics.”

As part of this added text, we included the following three additional references to inform readers of research that has been conducted elsewhere that may help address these limitations in future work:

49. Jiang M, Gao Q, Zhuang J. Reciprocal spreading and debunking processes of online misinformation: A new rumor spreading–debunking model with a case study. Physica A: Statistical Mechanics and its Applications. 2021 Mar 1;565:125572.

50. Wang X, Li Y, Li J, Liu Y, Qiu C. A rumor reversal model of online health information during the Covid-19 epidemic. Information Processing & Management. 2021 Nov 1;58(6):102731.

51. Gausen, Anna, Wayne Luk, and Ce Guo. "Using agent-based modelling to evaluate the impact of algorithmic curation on social media." ACM Journal of Data and Information Quality 15.1 (2022): 1-24.
